# Identification of Five Quality Needs for Rheumatology (Text Analysis and Literature Review)

**DOI:** 10.3389/fmed.2021.757102

**Published:** 2021-10-25

**Authors:** Johannes Pallua, Michael Schirmer

**Affiliations:** ^1^University Hospital for Orthopedics and Traumatology, Medical University of Innsbruck, Innsbruck, Austria; ^2^Fachhochschule Gesundheit, Health University of Applied Sciences Tyrol, Innsbruck, Austria; ^3^Department of Internal Medicine, University Clinic II, Innsbruck Medical University, Innsbruck, Austria

**Keywords:** quality management (QM), medical care, quality dimensions, health, musculoskeletal diseases, rheumatology, immunology

## Abstract

**Background:** While the use of the term “quality” in industry relates to the basic idea of making processes measurable and standardizing processes, medicine focuses on achieving health goals that go far beyond the mere implementation of diagnostic and therapeutic processes. However, the quality management systems used are often simple, self-created concepts that concentrate on administrative processes without considering the quality of the results, which is essential for the patient. For several rheumatic diseases, both outcome and treatment goals have been defined. This work summarizes current mainstreams of strategies with published quality efforts in rheumatology.

**Methods:** PubMed, Cochrane Library, and Web of Science were used to search for studies, and additional manual searches were carried out. Screening and content evaluation were carried out using the PRISMA-P 2015 checklist. After duplicate search in the Endnote reference management software (version X9.1), the software Rayyan QCRI (https://rayyan.qcri.org) was applied to check for pre-defined inclusion and exclusion criteria. Abstracts and full texts were screened and rated using Voyant Tools (https://voyant-tools.org/). Key issues were identified using the collocate analysis.

**Results:** The number of selected publications was small but specific (14 relevant correlations with coefficients >0.8). Using trend analysis, 15 publications with relative frequency of keywords >0.0125 were used for content analysis, revealing 5 quality needs. The treat to target (T2T) initiative was identified as fundamental paradigm. Outcome parameters required for T2T also allow quality assessments in routine clinical work. Quality care by multidisciplinary teams also focusing on polypharmacy and other quality aspects become essential, A global software platform to assess quality aspects is missing. Such an approach requires reporting of multiple outcome parameters according to evidence-based clinical guidelines and recommendations for the different rheumatic diseases. All health aspects defined by the WHO (physical, mental, and social health) have to be integrated into the management of rheumatic patients.

**Conclusion:** For the future, quality projects need goals defined by T2T based initiatives in routine clinical work, secondary quality goals include multidisciplinary cooperation and reduction of polypharmacy. Quality indicators and standards in different health systems will provide new information to optimize patients' care in different health systems.

## Introduction

Widespread attention to the quality of universal health care began in 2001 with a report by the Institute of Medicine (IOM) (1). This report identified the general public's interest, clinicians, and decision-makers, emphasizing morbidity and mortality due to medical errors. This report highlighted the need to restructure the health system to provide better quality and safer care. Accordingly, more and more attention was drawn to improve quality and safety in rheumatological health care in the past two decades. Rheumatology includes about 200 different, often rare diseases. In addition to this list of different diagnoses, the chronicity of most of these rheumatological diseases is also a particular challenge for care management.

Rheumatologists mainly focus on specific quality issues related to diagnosis and the effectiveness of therapeutic interventions. Adherence to national and international guidelines certainly increases the quality aspect of effectiveness and improve safety issues (2). Additional quality goals include patient safety, timely and patient-centered care, as well as effective, efficient and socially fair health care (1, 3–11). An expanded view of quality issues may also contribute to more attractive health care providers' work areas.

Many health organizations have developed quality indicators (QIs) to monitor, measure, and manage their health systems' performance and ensure effectiveness, efficiency, safety, timeliness, patent satisfaction, and equal access (12–14). Besides, a QI has to meet clinical relevance, scientific acceptance, and applicability (15) and be reliable and validated (16). Thus, QIs can independently reflect the actual performance of health organizations (17).

According to Donabedian's conceptual model, key QIs can be classified by referring to the three components of the health system's structure, process, and outcome. While the structure describes the context in which health care is provided (e.g., hospital buildings, staff, funding, and equipment), the processes encompass all transactions between patients and service providers throughout health care, and the results relate to the effects of health care on patients' health (18). All three components of this conceptual model should also be realized in rheumatology. In rheumatology, QIs for measuring the desired therapeutic achievements were used for both adults (19, 20) and pediatric rheumatology (21, 22). However, QIs and performance reports are descriptive and do not directly lead to any improvements in performance. Therefore, strategies are necessary for clinical routine, understanding the fundamentals of quality problems and practical tools to assure ongoing improvements of clinical outcomes. Accordingly, there is increasing interest in using electronic health records (EHRs) to develop and use electronic clinical quality measures (eCQMs) in rheumatology (23, 24). eCQMs are a new automated approach to extract information on QIs from the EHRs (23). Thus, in combination with data analyses from national clinical data registries, eCQMs not necessarily reduce the burden of collecting appropriate data, but they can serve as valuable tools for continuous quality improvement. Critical technological advances in building the infrastructure for the implementation of eCQMs will advance this field also in rheumatology (25).

It can be anticipated that outcome-oriented clinical quality management will become a standard procedure in the future and represent an essential pre-requisite to objectify medical competition—also in rheumatology. This work identifies and summarizes EHR-based quality needs for future efforts in rheumatology.

## Methods

A literature search was performed in the PubMed and Cochrane libraries as well as in the Web of Science search platforms in February 2021. The search was carried out using the following terms: “(rheumatology OR musculoskeletal diseases OR vasculitis OR arthritis OR connective tissue diseases) AND (quality of health care OR quality assurance health care) AND electronic health records.” In addition, a manual search was performed to identify additional potentially relevant articles.

English literature was included if published between 2000 and 2020. Non-peer-reviewed articles, case reports, comments, and conference summaries were excluded. A duplicate search was performed in the Endnote software (version X9.1) and the Rayyan QCRI software (https://rayyan.qcri.org) (26), together with manual screening of titles and abstracts based on specified inclusion and exclusion criteria. The literature screening and evaluation were carried out according to the PRISMA-P 2015 checklist (27).

The text mining software Voyant Tools (https://voyant-tools.org/) was used to read and analyze titles and abstracts. Analyses include a comparison of several documents, providing results from the Cirrus, the Collocates Graph, the Correlation, the Mandala and the Scatter Plot Tool together with a Trend analysis as follows (28):

- The **Cyrrus Tool** visualizes frequently used words of the documents as a word cloud, with words sized and positioned according to the frequency of their use. Color and absolute position of words are not significant.
- The **Correlation Tool** calculates the Pearson correlation coefficients by comparing the relative frequencies of terms (relative to each document for the corpus) (29). A coefficient that approaches 1 indicates that values correlate positively; approaching −1 indicates that values correlate negatively.
- The **Collocates Graph** represents keywords and terms that occur in close proximity as a force-directed network graph.
- The **Trend analysis** represents frequencies of terms across documents. Each series in the graph is colored according to the word it represents. At the top of the graph, a legend displays which words are associated with which colors. A cut-off of 0.0125 was chosen as relevant relative frequency for key terms, to be included into the qualitative analysis of the main quality issues.

The qualitative content analysis of the key quality issues for rheumatology was based on the trend analysis results.

## Results

A total of 1,251 records were included in the title/abstract screening of Rayyan QCRI (https://rayyan.qcri.org), including 21 records identified by manual research and after removal of 10 duplicates. Most of the articles came from the United States (*n* = 241) followed by the UK (*n* = 124), Canada (*n* = 69), and Australia (*n* = 61).

**Figure 1:**
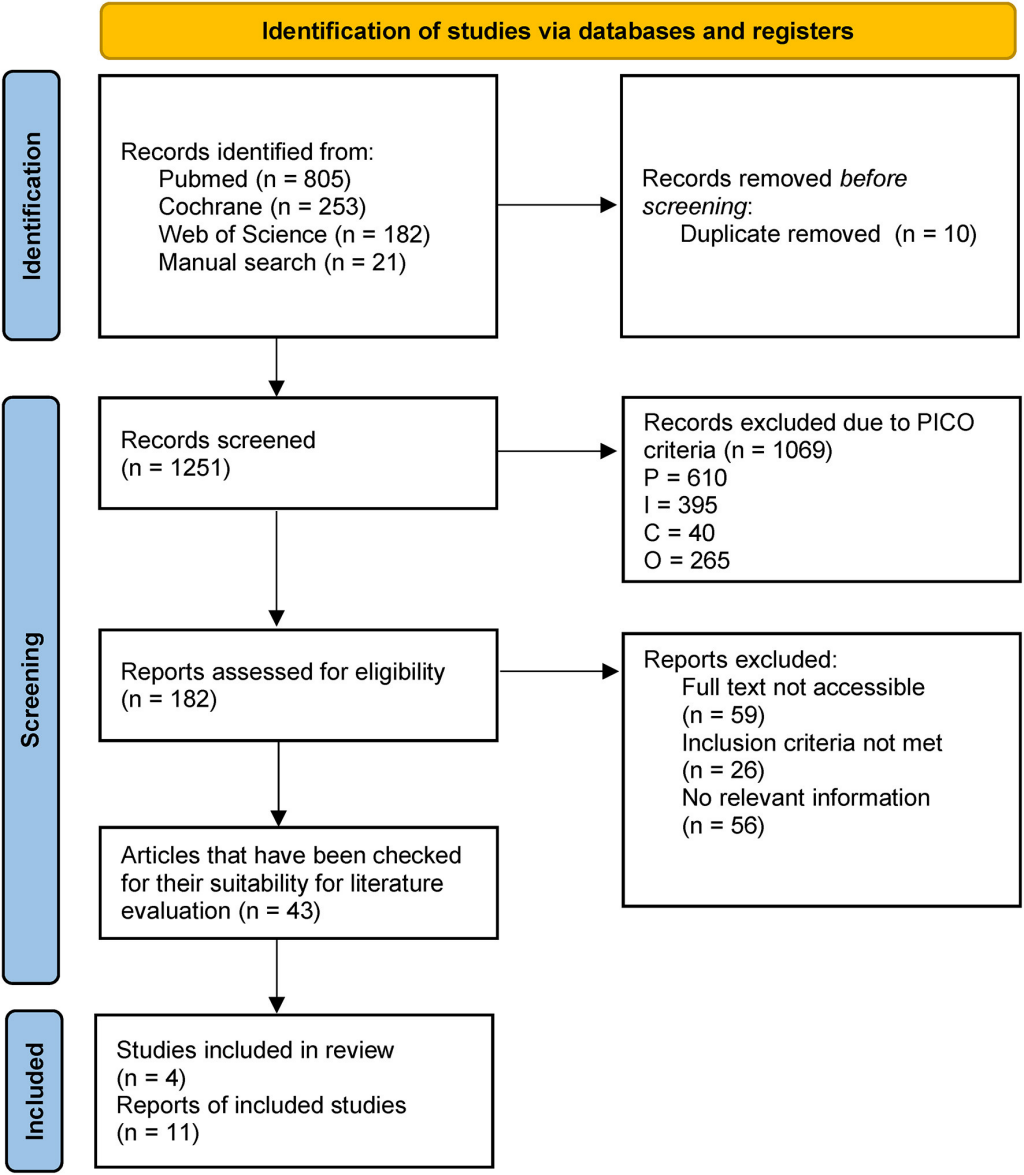
Flow diagram of records' selection according to the PRISMA-P strategy (27, 30).

Figure 1 demonstrates the flow diagram for the PRISMA-P strategy. One thousand sixty-nine records were excluded because they were irrelevant to the pre-defined questions, and 182 remaining full texts were checked for suitability using the PRISMA-P 2015 checklist.

### Full Text Analysis

Forty-three full texts (17 articles and 26 reviews) were included in the text analysis using the text mining software Voyant (https://voyant-tools.org/).

#### Cyrrus Analysis

**Figure 2:**
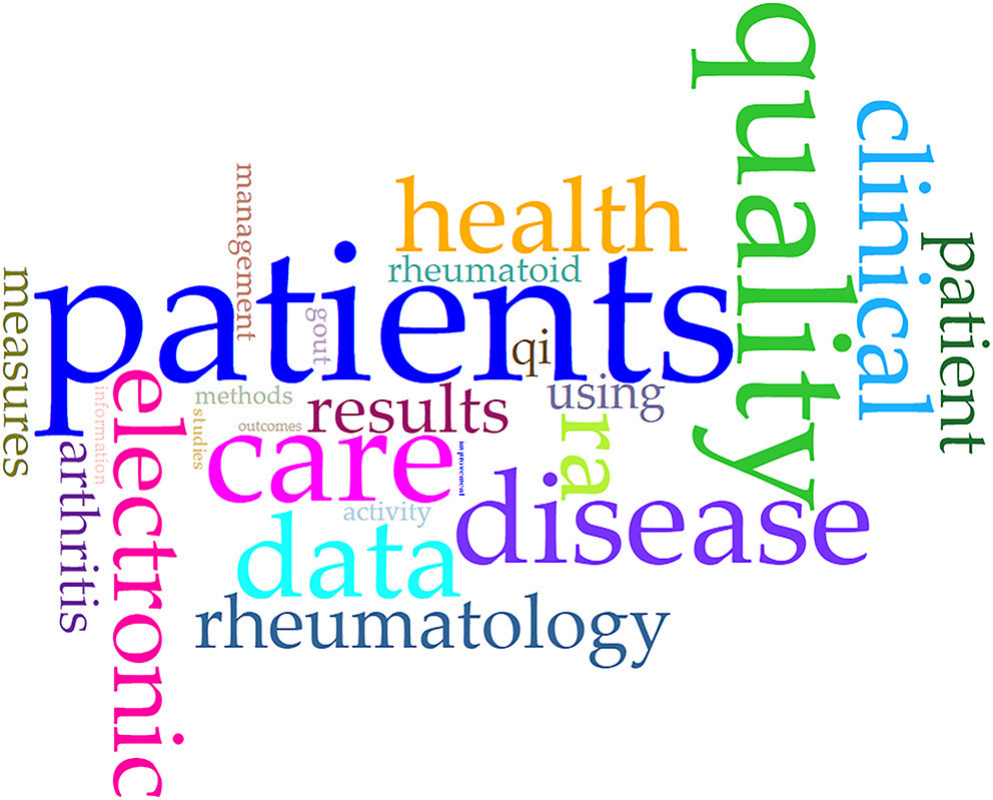
Cyrrus plot showing most frequently used terms of the literature search.

The most frequently used terms in the selected publications are visualized in Figure 2. Frequencies ranged from 142 to 33 per text file. This analysis shows that the literature search was carried out specifically for patients' care and quality (with 142, 89, and 108 nominations for patients, care and quality, respectively).

#### Correlation Analysis

Table 1 shows the Pearson correlation coefficients between the single-word citations in the selected publications. According to the search strategy, the strongest correlations exist between words related to the concept of “quality.”

**Table 1:** Pearsons correlation coefficients between single words of the literature search.

**Term 1**	**Term 2**	**Correlation**
Benchmarking	Treatment	1.0
Electronic	Quality	1.0
Indicator	Quality	1.0
Medical doctor	Quality	1.0
Arthritis	Electronic	0.9370426
Patient	RA	0.9370426
Improvement	Quality	0.8989332
Arthritis	Quality	0.8291562
Benchmarking	Quality	0.8291562
Treatment	Quality	0.8291562
Improvement	Quality	0.8291562
Indicator	Quality	0.8291562
Examination	Quality	0.80403024
Data	Quality	0.80178374

*A correlation coefficient that approaches 1 indicates that values correlate positively*.

*RA, rheumatoid arthritis*.

#### Collocate Graph

**Figure 3:**
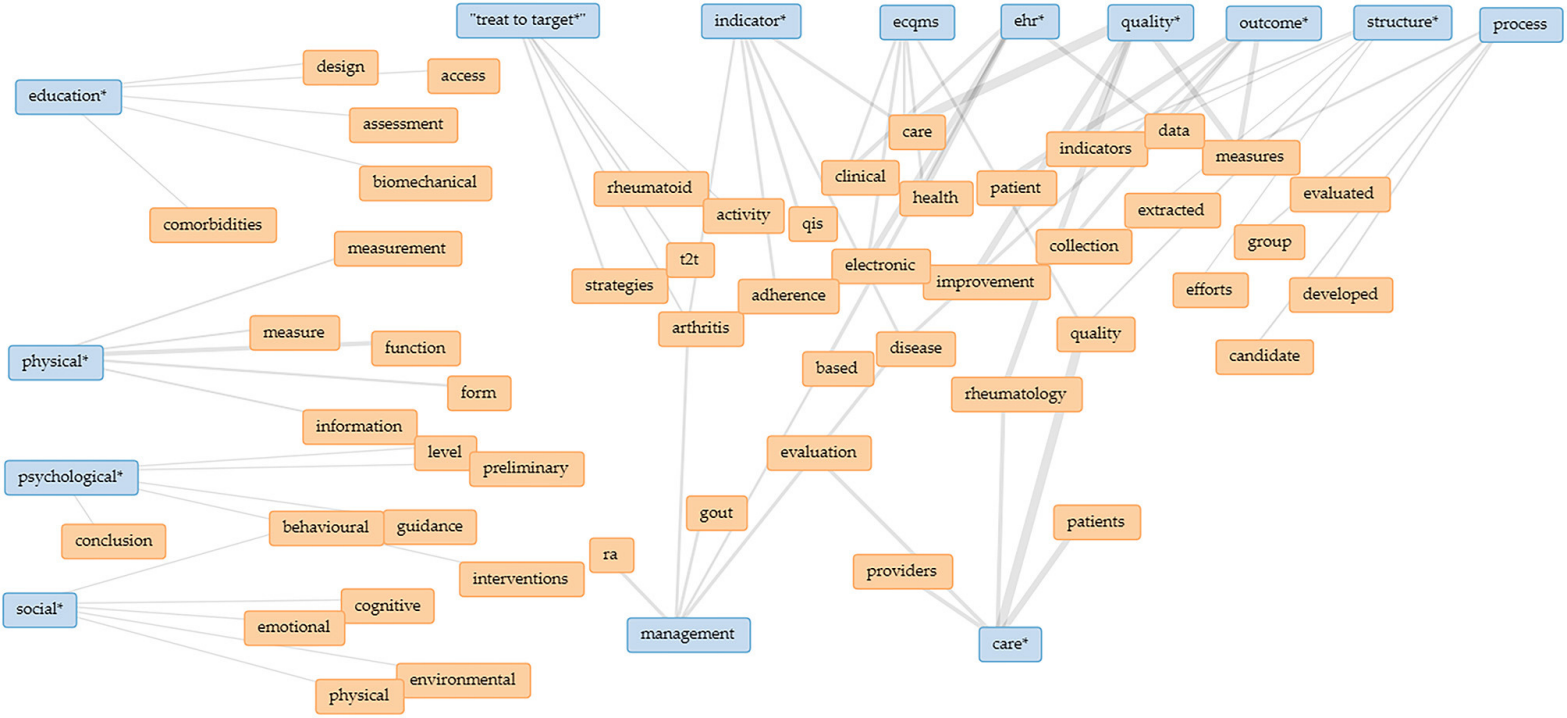
Network of terms identified by the Collocate Graph Tool. The group of “education,” “physical,” “psychological,” and “social” is clearly separated from the group of “management” and “care” as well as the group of “treat to target,” “indicator,” “ecqms,” “EHR,” “quality,” “outcome,” “structure,” and “process.” ecqm, electronic clinical quality measures; ehr, electronic health record; ra, rheumatoid arthritis; qis, quality indicators; t2t, treat to target. *including similar items.

The Collocate Graph represents keywords and terms that occur near a force-directed network graph. The relationship between various terms is shown as a network according to the selected literature in Figure 3.

This network analysis shows that according to quality-related literature the terms “physical,” “psychological,” and “social” (as used by the WHO to define the status of “health”), together with the term of “education” are separated from the remaining terms. Independent from this finding, a strong linkage exists not only between the terms “EHR,” “quality,” and “outcome,” but also with the Donabedian dimensions of “structure” and “process.” Even the terms “management” and “care” are rarely connected with “treat to target” so far. Based on this analysis, it can be assumed that there is a strong correlation between the quality dimensions in the selected articles, but links of these dimensions with the WHO-defined health goals and education could not be identified in the literature.

#### Trend Analysis

**Figure 4:**
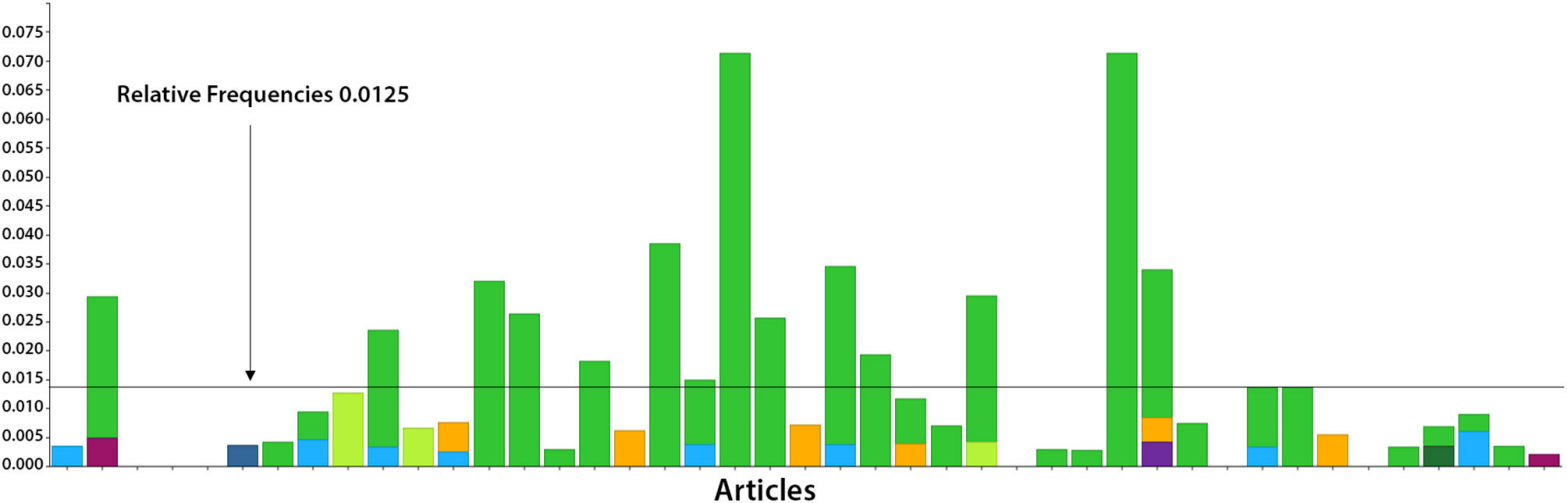
Trend analysis of the 43 full texts included related to quality (in green color), the Donabedian quality dimensions (“structure,” “process,” and “outcome,” shown in violet, light blue and orange color, respectively) and the WHO-defined health goals (“physical,” “mental,” and “social,” shown in light green, dark blue and dark green color, respectively). The cut-off of 0.0125 is shown as a line, indicating a relevant relative frequency.

The results of the trend analysis are shown in Figure 4. A total of 15 publications (11 articles and 4 reviews) showed a relative frequency of key terms which was higher than 0.0125 in the trend analysis.

Out of these publications, 1 article on structure (31), 7 articles on process (24, 32–37) and 2 articles on outcome quality (31, 38) were identified. For the WHO-defined health goals, 1 article on physical (39), no article on psychological and 1 article on social issues (40) were identified. One for the role of nursing (41), for polypharmacy 1 (42), and for treat to target (43) 1 publication was found in each case.

### Key Issue Analysis

The trend analysis for the content-related aspects identified five key issues for further content analysis. These key issues are outlined in the following paragraphs:

#### Quality Indicators for Clinical Routine

Measurements of outcome using validated outcome parameters are necessary to assess and further improve health care quality and have gained increasing momentum since gaps in the quality of care became evident in the USA and other Western countries (44). During the last decades, various tools were developed to improve health care quality like clinical practice guidelines, QIs and quality standards (QS).

A clinical practice guideline is usually defined as a systematically developed statement to assist physicians and patients in decision-making in specific circumstances according to best available evidence and/or expert consensus (45, 46). While clinical practice guidelines aim to improve consistency of care and compliance with evidence-based practice (46), compliance with clinical guidelines is not easy to measure (47).

QIs are specific statements that are often made in the following standard format: IF a specific clinical scenario occurs, THEN a specific action should be taken (48). A simplified example of a QI commonly used in rheumatology reads: “If a patient has RA, a disease-modifying anti-inflammatory drug should be prescribed” (2017). For other chronic conditions such as diabetes mellitus and high blood pressure, QIs are easy to use to quantify the quality of care. For these chronic diseases, the availability of a single measure as a gold standard makes it easier to measure the quality of care. However, in most rheumatic diseases, objective clinical, laboratory or imaging parameters alone cannot be used as gold standards (49, 50). Therefore, large datasets are required to identify an initial set of QIs separately for each rheumatic disease. A fascinating study was carried out with a huge sample of RA-patients from clinical practices in nine countries (51). The primary purpose of the database was to support clinical management and not to assess compliance with pre-defined QIs. The number of documented examinations differed significantly from country to country, which limited the comparison of compliance with QIs between countries. The main limitation of this study was that the database used was developed in 2007 before the QIs were created. The transfer of QIs between countries is difficult because of the geographical differences in clinical practice (31, 52). Another study addressed the implementation of structural, processual, and outcome-aspects of QIs to monitor rheumatoid arthritis (RA) (31). As this work was based on internationally published studies and guidelines, these authors suggested that only the “structural” QIs could vary worldwide due to different health systems.

In a comprehensive review of QS for inflammatory arthritis, significant heterogeneity was described for the methodology used to develop a QS (47). The methods ranged from expert discussions to formally modified Delphi methods, QSs were often proposed but not well-defined, and patients were not always involved in developing these QSs. Consequently, the authors recommended clear criteria for the development of QSs, as the introduction of all available standards is unlikely to be useful or necessary. In addition, the development of QSs should go along with the proposal of QIs to measure quality improvements. For adult spondyloarthritis (SpA), the international ASAS group of experts has developed a QS to improve health care services (37). These standards provide a clear description of the high-priority areas for quality improvement and monitoring. As part of this work, QS was formulated for the critical areas of referral, rheumatology assessment, treatment, education/self-management, and comorbidities. One of the authors' considerations was the transfer of this QS to other rheumatological diseases. However, as this QS refers to SpA and may not represent other rheumatological diseases, it is suggested that this QS is not directly transferred to other rheumatic diseases and can only serve as a template for other diseases. Unfortunately, the feasibility of this QS has not yet been tested in clinical practice anywhere in the world.

2. Treat to target (T2T) as a treatment strategy

The treatment target of clinical remission or at least a state of low disease activity was initially introduced into rheumatology for the treatment of RA, requiring frequent reassessment of disease activity and drug adjustments (53). Later on, the principles of T2T were also used for other guidelines (54–56). For the implementation process of T2T into clinical routine, audits and feedback loops are required (57, 58).

Implementation of the T2T strategy takes time, effort, and a good health system infrastructure, including platforms of EHRs, to support tracking of patients with follow-ups after specific therapeutic interventions. With subsequent audits and feedback, quality reports to rheumatologists can then initiate improved routines in rheumatology clinics.

Indeed, one initiative to optimize T2T strategies using audit and group feedback for RA provided deep insights into the implementation process: T2T resulted in a high measurement rate of disease activity recorded by an electronic platform, with suboptimal follow-up times identified for patients without remission, especially for patients with long disease duration (36). Three areas for potential quality improvement were identified during the group feedback session: (1) expanding the use of an electronic data collection software, (2) the need for a protocol to manage early RA, and (3) expanding similar initiatives to patients with other diagnoses. Limitations of this study were missing completeness of all outcome measurements, lack of relating outcome measurements to time from diagnosis to start of therapy (although the delay in treatment can affect the likelihood of remission or a state of low disease activity), and the single-center design (despite an excellent electronic infrastructure). For improved documentation of the actual medication consumption, the authors proposed a link to medication dispensing by pharmacies. Also, socio-demographic factors with the insurance status of the patients should be available.

Another study on the implementation of T2T examined the effect of collaborative training to facilitate the implementation of T2T, using the quality circles of plan-do-check-act (PDCA) (43). Designed as a continuous improvement model, phase 1 was a randomized clinical control study in which the intervention was compared with a control group, whereas in phase 2 the control group phased the jointly developed training intervention of phase 1. In contrast, the interventional group of phase 1 was further observed. During the PDCA cycles, T2T implementation was checked, but specific individual patient care problems were not observed. The strength of this joint training intervention was the collaboration between 11 different locations, facilitating the improvement and documentation of care, disease activity assessments, and treatment decision-making. Limitations were the high expenditure of time, checking the T2T goals being met, difficulties of generalizing documentation on the platform for EHRs and the location-specific nature of team meetings. This study confirmed that optimal RA management requires continuous monitoring of disease activity and continuous adjustment of treatment to maintain disease control.

There are several limitations to the T2T strategy: First, T2T requires process improvement with regular measurement of disease activity and timely adjustment of treatments, which is difficult without adequate support by cooperative EHR platforms. Second, in rheumatology, treatment goals are often defined using subjective outcome parameters, different from arterial hypertension and diabetes. This implies the necessary consideration of comorbidities, psychological and mental conditions possibly influencing the subjective assessment of disease activity.

3. Role of nursing in rheumatologic care

In several European countries, nursing has developed and specialized in the area of rheumatology (59–61). Specialized rheumatology nurses provide advice over the phone and offer self-management, education, and counseling for patients (62–67). In addition, they participate in disease management, monitor disease-modifying treatments, help treat comorbidities and, after special training, give intra-articular injections and prescribe drug treatments (59, 68–71).

In 2012, the EULAR recommendations on the role of nurses in the treatment of inflammatory arthritis were first published (72). The 10 evidence-based recommendations provided a basis for an improved and standardized level of professional care across Europe. The most important recommendations relate to rheumatological care availability, patient training, including self-management, evidence-based disease monitoring and management, patient satisfaction, and psychosocial support. In addition, three recommendations focused on professional support for nurses: availability of guidelines or protocols, access to training to take on more advanced tasks. The assessment of these recommendations showed a high degree of agreement between the countries, but there were significant differences in their application, suggesting that the implementation was not comprehensive (72–74). The EULAR recommendations were then updated in 2018 and expanded to include three new overarching principles relevant to all eight recommendations (41). This update confirmed the contribution of rheumatology nurses to health care, including telemedicine, thus offering new opportunities for accessible and sustainable health care (67). Person-centered care and partnership with patients are considered essential dimensions that promote trust in care (75–79).

4. Additional quality aspects like polypharmacy

The T2T strategy does not comprehensively include all quality aspects of managing rheumatologic patients. Polypharmacy is a typical example of an additional clinical aspect to be managed in routine practice, with possible need of quality improvement. There are several definitions of polypharmacy (80), including the number of drugs (usually over 4) and their inappropriateness (81, 82). Due to the different definitions, the number of patients affected by polypharmacy varies considerably (81).

Polypharmacy can lead to adverse drug interactions (83) and increase severe adverse events, thereby increasing healthcare costs (84). In elderly patients over 65 years of age, 50% will be prescribed more than 6 drugs, and nearly 20% will receive inappropriate medication (84). Comorbidities usually require additional treatment and may even lead to the prescription of unnecessary medication (85). So far, only a few RA studies have focused on polypharmacy in the literature: In a retrospective study on polypharmacy and comorbidities in Central European RA-patients, polypharmacy (≥5 drugs) was found in 34% of patients, especially in female patients over 50 years of age (42). This percentage is lower than 44.4–67.9% of all RA patients as reported in previous literature (86–88). Possible reasons are differences in the insurance systems or the definition of polypharmacy (82, 89). The main limitation is the retrospective design with incomplete data sets and the relatively small sample size of 175 RA-patients.

New to this study was the use of the international Anatomical Therapeutic Chemical (ATC) codes to classify drugs (42). This coding has not previously been used for the polypharmacy approach in RA, although ATC coding supports differentiation between disease-specific treatment and treatment of comorbidities and the study of additional pharmacological aspects during follow-up.

5. Data acquisition to provide quality indicators

Standardization of data acquisition is crucial for implementing quality efforts and applying QIs in daily routine practice. Although guidelines for data collection and outcome measurements have been developed (34), such initiatives have not consistently been implemented in RA (90, 91), and rarely in other diseases.

The principle feasibility of standardized data acquisition in routine clinical care is shown in Denmark (DANBIO) (92). Also, the Swiss Clinical Quality Management in Rheumatic Diseases (SCQM) project collects long-term follow-up data of patients with several pre-defined rheumatic diseases. Compared to these prospective approaches, retrospective data—independent from the technique used—should be considered lower quality data (93). In prospective approaches, any core data set implemented in registries, research cohorts, or systems should ideally balance clinical feasibility and the potential to generate valuable research data. For this purpose, a Delphi consensus-based checklist was proposed to provide data both for registries and routine clinical care (94).

As an example, a study systematically evaluated the American College of Rheumatology's (ACR) QI measures for monitoring RA and therapy with methotrexate (MTX) (32). For this purpose, six ACR-QIs were analyzed using the EHRs from several locations. Three ACR QIs focused on the management, and three ACR QIs focused on the use of MTX. As a result, EHRs supported easy tracking of laboratory values, but most EHRs could not collect and process information from both patients and physicians. The authors, therefore, suggested the use of validated software tools to collect and analyze this information, preferably in an integrated system as in the SCQM project.

## Discussion

Because of the many efforts to improve health care quality, it is difficult to identify key issues for quality initiatives. Indeed, there is a wide field of quality efforts in rheumatology, with quite diverse challenges to put the most important quality efforts into clinical practice. This work applied new text analyses in current literature, finally providing 5 key elements as quality needs for future initiatives.

First, the statistical text analysis using the new Voyant Tools (https://voyant-tools.org/) of titles and abstracts showed that the number of selected publications was small but related to the topic of interest—with high Pearsson correlation coefficients.

According to the Collocate Graph analysis of the same software, it appears that so far, the WHO dimensions of physical, psychological and social aspects required for the concept of health, together with the educational aspect, are not routinely linked to quality initiatives in the literature. This lack of coding literature according to quality issues can be even expanded to the use of the Donabedian model in rheumatology. Consequently, it will be essential for future searches to fully respect these issues, thus providing the information needed to address these quality issues in routine clinical practice fully.

The 5 key elements for rheumatology quality filtered out from the literature have to be discussed more in detail:

(1) QIs for clinical routine: This overview article does not offer any detailed knowledge about the implementation of QS in rheumatology, but it is necessary to point out the need for corresponding QIs, with which quality improvements can then be measured. Both QSs and QIs have to be validated (47) and include the needs and possibilities of those affected with the diseases and follow a pre-defined consensus process (94). Most available QSs and QIs were primarily developed in very well-developed health systems but should be used in all countries.
  Measuring QIs will help to close quality and supply gaps (32). Based on adequate QIs, the PDCA cycle then offers a practical approach to identifying and improving the quality of care (2, 95). For example, there is hardly any evidence available for some critical areas of quality improvement, such as the referral process. While the delay in diagnosis represents an essential gap in daily care, the challenge is that referral systems vary worldwide, and there are no indications of the feasible optimum of time (37). That is why the implementation and consecutive adaptions of QS in PDCA circles is crucial, and several components such as data sources, target group, and reporting period must be defined. The inclusion of different stakeholders (e.g., general practitioners, physiotherapists, patients, and pharmacists) can further increase the acceptance of such QS and QIs. Furthermore, focusing on the key areas of referral, disease assessment, therapy, education / self-management and comorbidities increases the likelihood of a significant improvement in the quality of care (37).
(2) Treat to target as treatment strategy: Despite the attractiveness of this strategy, it is not easy to translate it into routine clinical practice. In many cases, successful implementation requires structural quality with adequate human resources and process improvement with the definition of a suitable treatment goal with patient participation, regular monitoring of disease activity, and appropriate therapy adjustment (43). A good structural, personnel and technical infrastructure is required, including a software platform suitable for documentation and progress monitoring. The use of EHRs would support the implementation of the T2T strategy even better (96). A link with data on drug dispensing in pharmacies should also be sought in order to be able to better estimate the actual point in time of treatment changes. The results of audits and feedback could then be used to drive quality improvements (36).
(3) Role of nurses in rheumatologic care: Differences in the qualifications of rheumatology teams in different health systems determine the optimal use of the various professional groups and can also affect the quality of care provided to the patients (97). Indeed, the training and continuing education of nursing in rheumatology is not standardized in all European countries. Even the definition of “care” differs between countries. In the UK, for example, nursing has been defined as a practice model in which nurses, in collaboration with doctors and physiotherapists, occupational therapists and psychologists, provide education, monitoring and support for a specific group of patients (98). In any case, training should reflect the nurses' duties and activities (99).
  Nowadays, it is planned to align the level of competence across Europe. Initiatives to implement the EULAR-recommendations are essential and require national and international support from stakeholders such as the EULAR (41). Additional recommendations are needed that focus on other rheumatological diseases, including vasculitides. Together, these recommendations will further establish the importance of care in rheumatology and contribute to a standardized level of professional care across Europe.
(4) Additional quality aspects like polypharmacy: The application of drugs plays an essential role in managing rheumatic diseases. Polypharmacy is only one specific aspect, together with patients' compliance, number of comorbidities and monitoring of pharmacological side-effects during routine clinical management. Many of these aspects have not been well-researched and need further investigation (42).
(5) Data acquisition to provide QIs: For the future, EHRs should guide the relevant, diagnosis-specific QIs to be assessed quickly and easily in clinical practice (32, 51, 96). Such extended use of EHRs offers the possibility to evaluate clinical courses together with patients' perceptions of their outcome and perceived health care. Especially process- and outcome-related QIs have to be validated as instruments to provide transparency and help to standardize quality assessment (96).
  Indeed, health systems are increasingly using QIs directly extracted from EHRs. These measurements can then be used to evaluate standard care considering the outcome. Although electronic quality measures show promise in supporting to achieve potential goals of quality interventions, current studies suggest that significant infrastructure and analytical support at the practice level is necessary to integrate such electronic quality measurements into the existing health care systems (33). For rheumatic diseases with multiorgan manifestations and multiple disciplines, such standardized data acquisition should be carried out on an interdisciplinary platform.

At present, the user-oriented functionality of EHRs remains an obstacle, as cumbersome documentation, incorrect or repetitive data in drug lists and clinical notes, and lack of interoperability between systems of different physicians and health care providers can lead to reduced data quality. Expanding the collection of reliable data will result in improved reporting of rheumatologic quality (100–105), but requires user-oriented overall planning (96).

### Limitations

Since this work did not seek additional involvement from external stakeholders such as administration and insurance, the overview of evidence-based quality efforts in rheumatology was only addressed from the medical perspective. In addition, screening of literature by an independent expert would increase the objectivity of selected publications. Also, in the content analysis, strategies and application goals were summarized without details for their implementation.

Another limitation is that few studies were identified. This can be explained by the selection of the inclusion and exclusion criteria. Also, publications do not necessarily list possible links to quality aspects and refer to general medical problems more than to specific rheumatologic issues unless the work specifically addresses a certain rheumatologic disease. This aspect was taken into account by additional manual selection of important publications.

As a financial limitation of the overview, it should be mentioned that not freely available publications which were classified as being of little relevance due to their abstract were excluded from further review (*n* = 59). Thus, a limitation of the text analysis is that not all of the preselected texts were available in full-text format and therefore could not be analyzed. The generally recognized publication bias should be cited as a limitation, as it is more likely that statistically significant results will be published than studies that contain insignificant results or outline errors.

### Outlook

As conventional, unstructured routine documentation is—despite the possible use of automatic text search options—insufficient to address most rheumatologic quality efforts in clinical practice, future projects are required to standardize the documentation of all disease-specific QIs. It can be anticipated that using these QIs for direct patient care data acquisition will allow various future quality projects.

Although many approaches to quality improvement have been studied, the proposed interventions were subsequently not or only partially implemented in practice for reasons of practicality. This question, however, is of essential importance for further development of quality assurance in rheumatology and should always be addressed in parallel with the development of new quality approaches. While clinical studies usually follow an “isolated” approach with only one intervention for possible quality improvement over a manageable time frame, clinical settings vary in routine practice, and multidimensional approaches with long-term data are needed.

Quality efforts are currently carried out primarily in the in-patient area. It is essential for rheumatologic patients to comprehensively prepare and implement such quality efforts in an interdisciplinary manner and for the outpatient area. Aspects of the structure, procedural and outcome quality must be considered for in-patient settings.

Based on the findings above, future quality projects are needed, especially in the following fields of rheumatologic interest:

1. Initiatives to define the treat to target goals in all rheumatologic diseases:
  Definitions of T2T goals are necessary for all rheumatic diseases. Definition of disease-specific quality standards is decisive for implementing future quality efforts. It will be helpful to involve nursing staff, other medical disciplines and patients early in the developmental process, to enable implementation of quality efforts in the long term.
  The T2T goals can then be expanded to fulfill the health goals defined by the WHO.
2. Support of secondary quality goals such as avoidance of polypharmacy.
  At present, quality goals such as avoiding polypharmacy are challenging to achieve in clinical practice. However, since drug application plays an essential role in treating rheumatic diseases and comorbidities, both the number of possible side-effects and drug interactions can be examined to simplify patients' information. The use of ATC coding can certainly support such initiatives.
  Other “secondary” quality efforts will reduce the number of untreated comorbidities or lower the number of necessary accompanying operations.
3. Comparison of quality dimensions between different care settings (outpatient alone or with in-patient support) for different rheumatic diseases.
  Discussion about achievements of important quality goals will have to be conducted again and again. To acquire substantial evidence, EHR-based, rapidly updated, and disease-specific real-life data are needed to argue for quality-oriented strategic decisions. Thus, national and international comparisons between different institutions can contribute to the discussion of quality goals and assessments. Also, it will be possible to generate comparative data for individual disease management strategies in the future, thus contributing to the decision-making process for changes in therapy.
  We foresee that the advantages and disadvantages of different care settings will be decided based on evidence in rheumatology. The value of long-term outcome parameters will then gain more and more essential and can be argued with real-life data.

In the long-term, the strategy of future research projects will have to address all health aspects defined by the WHO (physical, mental and social health). Such comprehensive approaches may then support both the social and the mental health sector. Health organizations will often be asked about such comprehensive models of health care that meet the quality goals for the patients, defined by evidence from research studies.

## Author Contributions

JP and MS contributed to the design, performed the work, analyzed the results, wrote, and finalized the manuscript. All authors contributed to the article and approved the submitted version.

## Funding

Publication fee was funded by the Medical University of Innsbruck.

## Conflict of Interest

The authors declare that the research was conducted in the absence of any commercial or financial relationships that could be construed as a potential conflict of interest.

## Publisher's Note

All claims expressed in this article are solely those of the authors and do not necessarily represent those of their affiliated organizations, or those of the publisher, the editors and the reviewers. Any product that may be evaluated in this article, or claim that may be made by its manufacturer, is not guaranteed or endorsed by the publisher.
